# Gender Legacies of Jung and Freud as Epistemology in Emergent Feminist Research on Late Motherhood

**DOI:** 10.3390/bs4010014

**Published:** 2014-01-08

**Authors:** Maryann Barone-Chapman

**Affiliations:** School of Social Sciences, Cardiff University, Glamorgan Building, King Edward VII Avenue, Cardiff CF10 3WT, UK; E-Mail: Barone-ChapmanM@cf.ac.uk; Tel.: +44-20-8785-0043

**Keywords:** anima/animus, feminine/masculine, women’s psychiatric diagnoses, feminism, queer, creation myth, alchemy, union of opposites

## Abstract

While conducting doctoral research in social science on late motherhood, two analytical engagements with the feminine came to my attention as evidence of a patriarchal bias toward the realm of womanhood. Jung’s mythopoetic tension between symbolism and enactments with *the feminine* and Freud’s supposition that a denial of the feminine was necessary for psychological and emotional development appeared to be perpetuating a social problem continuing in current times. Across affective behavior and narrative within stories of late procreative desire, dream journals and Word Association Tests of eight participants was the memory of a male sibling who had enjoyed primacy of place in the parental home over the daughter. The female body with a voice was missing in the one-sided perspectives of Analytical Psychology and Psychoanalysis on the subject of the feminine, until a whole view of psyche’s discontents in Feminist inspired Psychoanalytic theories from both schools on the female body were included. Freud and Jung’s views became evidence of patriarchy as background while extension of Feminist inspired psychoanalytical thinking, Queer theories and Creation Myth allowed new meanings of the embodied feminine to emerge through a recapitulation of a union of opposites as a union of epistemology and ethos. The essence of Jung’s mid-life theories, altered by modernity and eclipsed by female advancement, remains replicatable and paradigmatic outside of essentialist gender performance.

## 1. Introduction

In the course of engaging with women’s stories and affects while exploring memories, dreams, and associations on the subject of delayed motherhood, two analytical ideas—Jung’s mythopoetic tension between symbolism and enactments with *the feminine* and Freud’s [1] “Repudiation of the Feminine” attracted my attention to the realm of womanhood as a social problem, in particular the way in which themes of psychic bisexuality produced a *feminine* that is “thereby displaced from its forced equivalence to the object and from its inevitable localization in the woman” ([2], p. 87). What kept coming up as both privation and deprivation across affective behavior and narrative among eight participants was the existence of a male sibling who had more privilege, encouragement and engagement with mother (and father if he was around) than the daughter. I realized these participants were demonstrating the very bones of this research, distinguishing the making of a complex between personal experience, cultural and collective contexts. The affects before me at micro level were emerging into a macro view of how feminism emerged when the feminine could no longer quietly accept being thwarted to favor the masculine. Like the Sumerian goddess Inanna, participants had taken their procreative desire underground until the clamor of mid-life beckoned them to reclaim the right to enjoy an ordinary life.

My aim in this paper is to examine the plural definition and uses of the feminine in Analytical Psychology and Psychoanalysis in particular against Western culture at large in order to define a Feminist ethos for this research. Though Jungian by qualification and perspective I must include my own reflexivity on theoretical problems such as the anima and animus in Analytical Psychology so that I do not unconsciously analyze the subjectivity of participants to Jungian or Freudian grand narratives on what it means for a woman to desire and experience motherhood in the fourth decade. But more so, not only does it appear the first analytical fathers offered us a useful theory of patriarchy [3] along with other documented effects of ‘the mind doctors’ on women [4,5], their androcentric frames of feminine reference becomes an important epistemology for delayed motherhood. *Female* diseases, such as depression, promiscuity, paranoia, eating disorders, self-mutilation, panic attacks, and suicide attempts, whether reported/treated or not, are all female role rituals ([5], p. 110) to which I’d like to add one more: the expectation of fertility after forty years of age.

## 2. Discovery Process

What is determined to be masculine and feminine behavior, expression, and choices continues in post Jungian psychotherapies as a question regarding development, even when these are attached to archetypes [6,7]. The biological difference in women with an implied imperative to reproduce opens the depth question of a woman’s unconscious use of her body as a means of separation, individuation and psychic growth ([8], p. 83). Delayed motherhood in a bio-technological age may be yet another form of power and control [9,10,11]. To consider late motherhood in a technological age begins with a review of Jung’s [12] early working through his ideas on the *contra-sexual other* of anima and animus, drawing from his real world experience of what a lack of procreativity means for a woman.

“…then you get into a special kind of hell…for a woman there is no longer any way out; if she cannot < does not > have children, escape into pregnancy, she falls into hellfire…she discovers that she is not only a woman, she is a man too”.

([12], p. 794)

Before the myths and terms of feminine and femininity are unpacked there is something very important to register about the finding of a favored male sibling in this research. Across all participants’ stories deep wounds to do with early gender learning of the superior value placed on the masculine in a brother, whether or not he was younger or older, while the good things of the feminine in the daughter were difficult to see by parental caretakers, were present. In effect these women had been groomed to feel inferior to the masculine, by being less considered, desired and entitled, resulting in a view they might be less capable in life than a male. That most of the eight participants enjoyed engagement in the world long past many of their peers due to onset of pregnancy around the fourth decade, goes some way to suggesting how their choice of delaying motherhood resonates, at minimum, with having to prove something to themselves and others regarding the very definition of what embodying the feminine is about; normative, predictive generative identity via motherhood was not going to be enough.

“The difference in a mother’s reaction to the birth of a son or daughter shows that the old factor of lack of a penis has even now not lost its strength. A mother is only brought unlimited satisfaction by her relation to a son; this is altogether the most perfect, the most free from ambivalence of all human relationships. A mother can transfer to her son the ambition which she has been obliged to suppress in her-self, and she can expect from him the satisfaction of all that has been left over in her of her masculinity complex.”.

([1], pp. 112–113)

The feminine principle equating to female inferiority by the founders of both Analytical Psychology and Psychoanalysis, appears along a continuum ranging from Freud’s perspective of causation, for example, his penis envy/castration theory was grounds for hysteria based on a phallo-centricity [2] to Jung’s invisible realm of the collective unconscious through the use of mythopoetics *as if* to rationalize logos as the sole propriety of men and Eros to women as a universal structuring element of psyche conceptualized as animus and anima, respectively. Jungian Analyst Polly Young-Eisendrath [13] frames these ideas as androcentric in their ignorance of the woman’s experience, her social context, and the nature of her female gender identity in context to traditional sex roles. Without conscious feminine experience “an anxious middle-aged woman, identified with the idea that she is inferior intellectually, may be called ‘animus-ridden’ by a Jungian psychotherapist because she speaks in an opinionated and insistent manner about a general or vague idea” ([13], p. 23).

### 2.1. Feminine Riddles into Myths

Image, emotion, enactments, projection, rituals and fantasies emerging as beliefs in early Psychoanalytical theories reify mental phenomena, blurring the lines between illusion and reality. Jung and Freud appear as early social scientists looking to explain the split between matter and mind. Once Freud’s descendents opened the gate to allow for the impact of culture on phenomena observed by the analytical founding fathers, the groundwork was laid for Feminist inspired Psychoanalysis to evolve into psychosocial research, including embodied subjectivity. “For example, for Lacan, the Oedipus complex becomes not simply the exclusion of the child from the mother-infant dyad and parental couple which is thought by Freudians to be crucial for developing personality, but more a depiction of the beginning of the *acculturated individual*—that is, the entry into, and the reproduction of, *culture itself* repeated in the development of each human being” ([14], p. 294). Culture reproducing itself also extends to mothering [15]. What follows is the effect these analytical ideas can have on society.

“…some psychoanalytic concepts have taken on the quality of myths. I define myths as symbolic representations of cultural ideologies, reflecting unconscious dynamics. As with individuals, sometimes stale and outgrown myths persist, sustained by inherent societal forces even beyond their point of usefulness, resistant to change and often obstructing growth and creativity. Most psychoanalytic concepts originate as explanatory hypotheses. However, once formulated and disseminated, they become rooted both in theory and in society, acquiring an explanatory force, generating self-fulfilling prophesies and remaining unchanged as long as the myth serves a purpose…even when there have been changes in phenomena upon which the initial observations were made, the original hypothesis, reified and elevated to the proportion of a myth, remains immutable, sustained for the social, economic, political or psychological purpose it now serves.”.

([16], p. 8)

Though Freud is credited with asking the question, “What do women want?” he never found an answer to the “riddle of femininity” [16] and neither did Jung except through personal foibles [17]. The favoring of Jungian Psychology I had intended for this research was discovered to be insufficient to reflect on an emerging cultural problem with the feminine. There was danger of falling into Jung’s earliest reifications of gender on archetypal and functional levels underpinned by his interest in alchemical processes of the solar king meeting the lunar queen ([18], pp. 282–284). Jung’s ([19], para. 4–46) identification of two kinds of thinking along gender lines of masculine and feminine, classified as “direct” and “indirect” (feeling) thinking, is a case in point where early psychological typology function is confused with gender function. Indirect thinking was deemed to be intuitive, irrational, pictorial, diffuse and symbolic. Jung assumed it was the foundation of feminine psychology ([20], p. 54) under the principle heading of Eros, to include psychic relatedness, love and soul which also put women under pressure to perform as such in the activities of wife, consort and mother. Direct thinking, logical, goal oriented, rational, differentiated, and spoken skills, gathered together under the principle of Logos became the expectation of the masculine principle and ergo for men. Jung assigned words like judgment, discrimination and insight as well as spirit to ‘maleness’ ([19], para. 87).

My sense of Jung is that he read into the reproduction of gender performance and culture *as if* his identification of its’ contents was fact, confusing fears and fantasies with real women [13]. Not all post-Jungians read gender the way he did, but of those women clinicians presenting themselves as Jungian Feminists, such as Cowan [17], Douglas [6], Kulkarni [21], and Anthony El Saffar [22] few other than Young-Eisendrath [23] are known and published within the larger context of Psychoanalytically inspired feminism, I believe, because she draws from social constructivism to assert the ‘feminine archetype’ which is a product of patriarchy [24]. Yet Kulkarni [21] was among the first to lay down a paradigm for a research that “marries Jung’s respect for psyche with feminism’s insistence on context” ([21], p. 218), an ethos this research on late motherhood endeavors to achieve. In addition, two academics, Demaris Wehr [25] and Susan Rowland [7,26,27], have made breakthrough and remarkable contributions. In particular is Rowland’s ([7], p. 135) view of Jung’s connection to feminism through his concept of the subtle body, a union of mind and body in his alchemical writings, which includes “the abject and excluded body to reveal it as the constituting boundary of heterosexuality that must be renegotiated” ([7], p. 144). In a parallel but different language, de Beauvoir’s “One is not born, but becomes, a woman” ([28], p. 301) was a favoring of lived experience which inspired emerging feminism to make the distinction between sex and gender, an idea meant to “secure internalization of contrasting patterns of behavior… thus to displace the role of biology in determining ‘masculinity’ and ‘femininity” ([29], p. 39).

Psychoanalytical theorists have gone further than Freud’s ideas of the feminine, contributing to and developing Feminist theory aligned with clinical and social psychology theorists. Raphael-Leff’s [30] inquiry into femininity, the unconscious, gender and generative identity in a bio-techno age argues that a basis of psychoanalytic theory in place throughout Freud’s life was the limitation of femininity and masculinity on original bisexuality. The perception of Freud’s bisexual fluidity concept was ultimately eroded by occluding “reification of body-based dichotomies” ([30], p. 500) leading to multilayered views of fantasies/relational configurations/identifications proffered by Harris [31], Dimen [32], Benjamin [33], and Sweetnam [34] allowing Raphael-Leff [30] to frame Freud’s notion of bisexuality as the dichotomy of conscious unity twinned with unconscious diversity attributable to Person [35], based on Goldner’s [36] notion of culture as authorizing agent. Thus Raphael-Leff’s ([30], p. 501) synthesis of ‘sex’ as an accommodation between chromosomes present at birth and gender as a self categorizing psychosocial construct produces new categories for ‘gender role’ and ‘sexual orientation’: “‘*Embodimen*t’ (femaleness/maleness), ‘G*ender Representation*’ (femininity/masculinity) and ‘*Desire*’ (sexuality).” Can Jungian Feminist literature ever be on par with the impact Psychoanalysis has had on mainstream feminism? Jung’s dichotomous idealization of the feminine as a man’s anima while denigrating the masculine in a woman (animus) as a character flaw, at first blush creates a problematic for the researcher who wishes to use Analytical Psychology as the theoretical basis for emergent *feminine* Feminist psychosocial dilemmas, until we shortly come to discussing his alchemical works. Jung’s mythopoetical views, theories, imaginations, foibles and proclivities regarding the feminine, along with Freud’s fluid notions of bi-sexuality, are both offered as evidence; acceptance of the feminine as different but equal remains a long standing difficulty for both genders, inspiring perhaps the intra-psychic and inter-subjective cultural phenomena of a *pregnant pause* [37] on the way to late motherhood, to revision the feminine out of patriarchal paradigms. 

### 2.2. The Feminine and Feminism

By emphasizing the *feminine within* feminism, I am including ways of incorporating agency and nurturing through the holistic union of Jung’s two kinds of thinking [19] in addition to Feminist concerns of equality with men such that *procreative identity* does not become equated to essentialist gender norms nor to performance in male terms. Holding on to the *feminine within feminism* allows for sexual difference and keeps in mind the ways the *feminine* has long been suppressed in culture [22], her wound the subject of myths and fairy tales ([38], pp. 193–194). Without this view it would be all too easy to see women who fell into delayed motherhood as ‘father’s daughters’ who abandoned the archetypal feminine to pursue career rather than respect the body Marion Woodman [39] likens to the Mother in us. What happens to women who like Inanna must go underground with their procreativity is far more complicated than being ‘father’s daughter’. Late motherhood does not appear as a sin against the feminine by the woman who has delayed, but as a ‘repudiation of the feminine’ preceding adult choices necessitating a late search for the mother within. Hence feminism and the feminine as Great Mother is a vital link to re-balancing humankind.

While aspects of Analytical Psychology are relevant to this study *Feminist inspired* Psychoanalytic perspectives help to make two halves of analytic history a whole view of psyche’s discontent with patriarchal views of the feminine. Analytical psychology has a proud history of finding truth in the cosmos through archetype and image “rooted in the unconscious as transcendent of knowledge” ([7], p. 143) while Swartz reminds us that “Feminism has a proud history of interrogating the truth claims of psychiatric science, and of foregrounding the ways in which the machinery of psychiatric diagnosis and treatment has been used to obscure or amplify the psychological effects of patriarchies” ([9], p. 41) for which she credits Chesler [5], Smith [40] and Ussher [41]. In particular, in reviewing psychiatric diagnosis from a Feminist perspective, Swartz ([9], p. 41) gives credit to Jessica Benjamin’s [33] work concerning the long history of patriarchal domination where Feminists have challenged Freudian psychoanalytic diagnostic premises and opened up new ideas on the formation of female identity such that experience as mother, sister, wife, or daughter can no longer be automatically synonymous with a lack of agency. My purpose is not a rapprochement between Jungian and Freudian theorists and clinicians, but observation early views of Jung and Freud on the feminine provide grounded evidence their theories continue to reflect a problem for and with women.

Given the nature of this study, to explore delayed motherhood and its connection to individual and collective complexes, and the long history of women being diagnosed as “prone to depression” ([9], p. 23) it is important to clearly differentiate the identification of a complex from a diagnosis. In a diagnosis the root of the disorder is placed within an individual while social, cultural, political and collective contexts remain as background or in ignorance [9]. Delayed motherhood in the 21st century begins to appear more as an emerging ‘epidemic’ with plural longitudinal gender roots between the sexes [37] rather than a disorder (though it may have been viewed so by Freud and Jung at one time). Identifying a complex through the study of affective behaviors provides a way to see into emotional rupture as phenomena, which does not originate in the individual alone, but through a network of associations involved in memories with others. These ‘others’ do not only contribute to personal complexes, as they may be unknown to the individual, because they occupy a place in the social through the cultural unconscious [42,43]. When these impersonal contexts are included in what happens when a woman is unconscious toward her body, we must consider the feminine in context to patriarchy, and by extension Feminist ideas. It must also be noted that patriarchy does not always have a penis, nor do Feminists always come with a vagina, and shortly I will elaborate on this further. 

## 3. Defining Problems

Both Analytical Psychology and Psychoanalysis have framed woman as subject, object, abject, Mother, other, caregiver, mirror, animus ridden, anima woman, receptive, castrated, empathic, relationally oriented, envious of a penis, a uroboros for renewal and imaged as the contra-sexual unconscious. When the female is not referred to as part object and part symbol, we find a purpose for her existence as “another subject whose independent center must be outside her child if she is to grant him the recognition he (she) exists” ([44], p. 24). The use and relationship to the ‘feminine’ in all its variations, including ‘femininity’ emerged as the ‘last straw’ turning Freud and Jung from sparring partners on ‘universal principles’ to ‘warring opposites’. Both men were caught in the prejudices of patriarchal culture to do with rights, roles and conduct of women in relation to men, pleasure and becoming a mother, until the mother-son incest taboo provided grounds for their ultimate parting of ways ([22], pp. 46–47). 

The difference between sparring over the existence of an underlying universal principle and the mother-son incest taboo may seem to be intellectually far apart until we discover how each of these men interpreted their necessity. For Jung mother-son incest functioned as a mythopoetic in intra-psychic life. It was seen as an enactment within his counter-transference dynamics with patients, such as Spielrein, while his wife Emma and consort Toni Wolff, and collection of female colleagues known as the Jungfrauen*,* all allowed him to be convinced “that the father’s law against incest is regularly broken on the symbolic level, and that regression to the womb is also part of the hero’s journey to rebirth” [22]. Whereas in Freud’s [1] thinking a girl’s cure for narcissism is not only founded on the discovery she does not have a penis, but on the move from mother to father to husband where her triumph and cure is the production of a son with whom she can “transfer to her son all the ambitions she has been obliged to suppress in herself…” ([1], p. 133). Freud’s thinking is a natural wellspring for feminism. While Jung’s psychology continues to entice women into believing they could be a man’s muse and *inspiratrice*, just as Echo helped Narcissus to continue looking at his image, believing it to speak to him in his favor [45].

One of the first Jungian Analysts to question the masculine psychologies of Jung and Freud, James Hillman ([46], pp. 291–292), finds in Freud ([47], p. 219) a definition of the conditions under which an analysis may end, based upon the achievement of “feminine inferiority’, finding it to be ‘the root of repression and neurosis… bringing about both our psychic disorders and method of analysis aimed at these disorders” [46].

“…one reaches the ‘bedrock’, the place where analysis could be said to end, when the ‘repudiation of femininity’ both in a man and a woman has been successfully met. In a woman the repudiation of femininity is manifested in her intractable penis envy; in a man his repudiation does not allow him to submit and be passive to other men”.

([47], p. 219)

Thus for Hillman [46], Freud’s [47] “repudiation of femininity” is biologically founded and part of the natural psychical world in contrast with his own view “the end of analysis coincides with the acceptance of femininity” ([46], p. 292). Here Hillman takes on misogyny by undermining Freud’s basis as “biologically given and thus ‘bedrock’ to the psychical field” ([46], p. 292), finding instead a psychological basis of ‘Apollonism’ as the ‘bedrock’ of the “first-Adam-then-Eve” perspective. This Apollonic archetype seeks physical form through “an objective and detached selfhood, a heroic course of… quest and search… above all the ego-Self as its carrier, and analysis as its instrument” ([46], p. 293). With Freud we must put aside the feeling and relational aspect of the feminine; biology rules. Re-creation of the myth ‘first-Adam-then-Eve’ appeared in the earliest memories of research participants in the triangulation with parents and male siblings. As young women, they purposely chose to use their minds and make non-uterine choices tending to put them more in the world of men, such that the structure of their lives begins to suggest an extended Apollonic phase. From just this small glimpse into Freud’s thinking of the feminine through one of his last writings in Vienna, it may be possible to see the necessity of Feminist thought to salvage Psychoanalysis from Freud’s complaint “psychology cannot solve the riddle of femininity” ([1], p. 149). 

For Jung the analytic process reaches its ultimate goal in conscious bisexuality through the alchemical image of the coniunctio/the conjunction [46,48,49]. Rowland [7] redeems Jung for Feminists in analyzing his work as a whole, and in particular on alchemy where there is “recognition of the limitations of heterosexual opposition… what is cast out, what is structured as an abject body, must be reconfigured *within*” ([7], p. 145). This is the maddening aspect of Jung, saddling Analytical Psychology with his biases of appropriating the feminine as a hidden virtue of men with the anima concept only to find him projecting onto women the worst attributes of the masculine with the concept of animus, opposite and not equal yet destined for bilateral unity. What is required here is a slow careful reading of Jung as a *trickster* [27] writer to be read for multiplicity as an evolving narrative rather than authority [26]. “Jung’s writings are characterized by an entwined dual purpose in which an acknowledgement of the roots of his ideas in his individual experience (personal myths) work with, and against, a drive to universalize and construct a comprehensive psychological scheme” ([26], p. 25). Nowhere is this more evident than in his move from the oppositional neurotic on gender to alchemy’s subtle body and external reality to social discourses ([7], p. 145). Samuels [50] questioned whether Jung’s concept of anima and animus/femininity and masculinity, entwined in the syzygy to endure the alchemical processes of differentiation in an effort to re-unite as an androgynous pair of opposites, was a bonafide work on gender. “Jung often spoke as if he were unaware of the distinction between gender and sex, which is, by contrast, biologically determined” ([20], p. 60). The feminine as an aspect of men and the masculine as an aspect of women became tangled up in Jung’s reflections between biological bodies, the embodiment of archetype and effects of culture and the collective unconscious. This is no different to what happens to anyone when the principle of ‘masculine’ and ‘feminine’ is concretized as first Adam then Eve. A false adaptation to compensate for psychic wounds to sexual identity, aroused by conformity to cultural stereotypes can sublimate the feminine such that men find they want babies and women are afraid to have them [49]. When the feminine in either gender is denigrated things go wrong, a link to the alchemical subtle body becoming physically and psychically blackened, precipitating a sulfuric decay to rise so that the problem as it is felt can dissolve [49]. 

In Feminist inspired Psychoanalytical literature longitudinal consideration has been given to self-images of feminine and masculine internalized through separation-individuation rituals within family as part of an evolving acquisition of gender-role identity commencing with “differential permutations of mother/father-boy/girl interactions, with the ‘feminine’ situated in the historical fact primary caregivers were invariably women” ([30], p. 503). Raphael-Leff [30] offers the observation of mother frustrating dependency, thus becoming the confusing feared and desired catalyst for counter denigration of all that is designated female [51]. In Rapahel-Leff’s view it is the mother that carries reproduction of the patriarchal social order of inferior social position, through unconscious same-sex identification with their daughters [30]. This identification can be seen later in threats to reproductive body integrity [52], preferred female relatedness [53] and an ego with porous boundaries like a mother [54] compelling a daughter to give into/ resign herself to the patriarchal social order [3,55]. 

### 3.1. Confounding Gender

It is essential to return now to amplification of Jung’s alchemical opus, as a psychic process which involved extracting the gold and liquefying the dung within primal matter, including elevating the ‘opposites’ to the regal status of Sol King (conscious) and Luna Queen (unconscious). Appearing in every culture, these motifs were intuitively drawn over millennia to signify psychic renewal, forecasting how dominant factors in the psyche undergo processes of decomposition and clarification by fire, out of which emerges the ‘new king’ or new consciousness [49]. This alchemical process may also serve as a paradigm for developmental processes within the *pregnant pause* of midlife [37]. The emergent new conscious of desire for a baby becomes the new king after years of licking the wounds inflicted upon the feminine within procreative possibility due to modern cultural conditioning to favor the masculine over the feminine for economic performance. Thus women’s lives take on the appearance of a two-part structure: first Adam then Eve. This is perhaps the basis of Jung’s division between the Logos of a monotheistic God whose “essential separation from nature sponsors rationality as dependent upon a division from matter and body” ([56], para. 29, 41) and the need of Eros to be connected and related as the Mother Earth [28]. “Jung’s early disposition for gendering opposites, with varying degrees of denigration and idealization, though evidence of extraordinary early work on identifying contradictions in nature seeking reconciliation” [49] similarly to Freud, appears to be reinforced by the mythopoetics of misogyny and female inferiority in the collective unconscious ([46], pp. 215–298).

“Jung’s entire project, I am suggesting, is, in mythical terms an attempt to re-balance modernity that has been brought to crisis by an over-valuing of Logos at the expense of Eros-relating…by essentializing the creation myths, he is able to stabilize the masculine signifying he wants to retain it, while insisting upon its re-formation to include the feminine, which remains marginal”.

([27], pp. 290–291)

### 3.2. Queer and the Feminine Hero

Queer theory emerges in personal identification and political organization as non-normative performance in a range of experiences of being and doing, inspiration for intra-psychic unions where achieving and nurturing, penetrating and receiving, are un-assigned to gendered bodies but co-exist in any body [49]. Citing Queer theorists Elizabeth Freeman and Judith Halberstam, Emanuela Bianchi [57] presents a movement “From Feminine Time to Queer/Feminist Time” ([57], p. 41) to notice how temporality in Queer strays from the normative, “unaccountable and dilated time” ([57], p. 41) arguing that pregnancy and mothering both participate in temporal counter-normativity. When viewed as a formulation of ‘women’s time’ with “women’s characteristic capacity to be interrupted, by the demands of family, by pregnancy… we take into account the necessity for protecting against hostile and unwanted interruptions *as well as* promoting a liberatory trans-valuation of interrupted time… to strange new, queer formations of kinship, gender, and social life” ([57], p. 43). When gender performance enacts a great leap of faith outside of predictive maternal identity as biological destiny, late motherhood, as I have found in participants’ case studies, is the struggle to achieve and nurture, penetrate and receive; a modern developmental task for the feminine hero. Theoretically, “the androgyne, a union of masculine and feminine which cannot be defined as either, resisting normative gender identity, is the essence of Queer. Understood this way, Queer is in effect the conclusion of Jung’s alchemical opus, the Philosopher’s Stone” [49]. 

The assumption of heterosexuality and gender certainty is a problematic of classical Jungian canon. Despite my and other Jungian Analysts’ criticisms of ‘gender certain’ contra-sexual opposites, the archetypes of anima and animus, continue to appear in dreams to reveal shadow aspects, those parts of the self that are unknown, unwanted and un-integrated, as principles of both *agentic* and *allowing* energies seeking conscious integration in men and women. To dismantle gender performance from procreative identity and sexual desire was a pre-requisite for analyzing the embodied feminine as she coursed her way through intra-psychic association networks and inter-subjective affects aroused by the methodologies used in this study.

Recognizing “the effect of the patriarchal animus on generations of women” ([6], p. xviii) Jungian Analyst Claire Douglas examined the outmoded aspects of Jung’s theories including the ephemeral, contaminated, and biased, to find what would free women, and the feminine from patriarchal precepts. She proposes a re-examination of the words and ideas within ‘Jung’s map’ rather than conforming to concretized descriptions as normative. “The feminine ego needs to learn how to connect without being engulfed, and how to differentiate without severing or splitting off” ([6], p. 299). Where Douglas’ thinking can be most readily applied is to the idea that the masculine as animus must reside solely in the internal world of the woman, and for men the feminine anima must stay safely locked inside. While I do not question the psychic reality of these figures, identification of what is anima and animus has an unfortunate link to opposite sex gender in a straightjacket of inferiority. Anima and animus need each other in dialogue, taking turns as sources of authority.

Gray [58] set out to examine, in philosophical terms Jung’s *individuation* idea next to the subject of the feminine by drawing from Irigaray’s work.

“Individuation, I claim, is the telos of Luce Irigaray’s ideal of a feminine-feminine symbolic/imaginary or system of meanings and significances that arises out of sex/gendered embodiment and collective responses to it…lest this reading of Jung be interpreted as reinscribing masculine notions of the feminine, I take a new look at the idea of essentialism, which has plagued Jung’s own theoretical construction of the feminine and ‘woman’…and also Irigaray’s approach to the woman question”.

([58], p. ix)

Jung perhaps explains his gender biases best in describing his view of opposites in male and female terms followed by problems when the opposites are not in their ‘right order’.

“…woman’s conscious is characterized more by the connective quality of Eros than by the discrimination and cognition associated by Logos. In men, Eros… is usually less developed than Logos. In women on the other hand, Eros is an expression of their true nature, while their Logos is often a regrettable accident”.

([56], para. 29)

“…instances to the contrary leap to the eye: men who care nothing for discrimination, judgment and insight, and women who display an almost excessively masculine proficiency in this respect… Wherever this exists we find a forcible intrusion of the unconscious, a corresponding exclusion of the consciousness specific to either sex, a predominance of the shadow and of contra-sexuality”.

([59], para. 225)

In her chapter on the ‘Feminine Hero’ in *The Presence of the Feminine in Film*, Jane Alexander Stewart [60] analyzes the role of Clarice Starling (played by Jodie Foster) in *The Silence of the Lambs* [61] as a “new heroic journey of the feminine” ([60], p. 95). Clarice’s story in the film begins with her lifting herself out of a chasm to stand at the top of the hill prepared to go forward. Stewart makes meaning of the scene in that “Clarice begins her story where classic stories of the heroine’s journey end; at the return to ordinary life after the descent… from a metaphorical feminine center…a heroine making a return from the deep process of self examination and affirmation” ([60], p. 96). Though the context of her meaning making resides in the modern American landscape where unseen killers await, her real message is not so much based on geography but an endemic fear of psychological and physical denigration of the feminine.

“Not only do they fear men’s attacks on their bodies but also they face denigrating social systems that reinforce a second-class status and devalue what it means to live through a feminine point of view”.

([60], p. 96)

These dangers, horrors and defilements have been described and examined by both Kristeva [62] and Douglas [63] within a frame of prohibitions leading to abjection on a platform of incomprehensible fear for the dangers facing the feminine if it is not pure. With Clarice Starling we get a character who succeeds because she manages to claim and hold fast to her feelings, what Alexander Stewart refers to as “a set of feminine ethics… [to]… create hope for the safety of a feminine presence in our society” ([60], p. 96). Clarice defies conventional wisdom on what is safe for a woman in a man’s world, by not behaving like a man who fears for his survival. Instead Clarice chooses to trust what the feminine has to offer, “her inner forces (for example trusting in intuition, in revealing herself and interacting on the level of intimacy)” ([60], p. 99) traits that invoke fear for her and of her, a greater threat to her survival than Hannibal Lecter himself, including “searches for meaning from the way his actions make her feel” ([60], p. 104). 

Citing Barbara Walker’s [64] *The Woman’s Encyclopedia of Myth and Secrets*, Alexander Stewart ([62], p. 103) offers an image, not only of the filmic style of Demme’s *Lambs* to evince the underground, underwater, under-position of Starling’s journey, but an insight into the journey toward motherhood in the fourth decade of life.

“Students in mythology find that when the feminine principle is subjected to sustained attack, it often quietly submerges. Under the water (where organic life began) it swims through the subconscious of the dominant male society, occasionally bobbing to the surface to offer a glimpse of the rejected harmony”.

([64], p. 1066)

## 4. Discussion

The feminine hero may be different from the heroine in my observations. The heroine comes up in life believing it is safe to be female because her nurturing early environment made it so. Throughout her development she does not cower at real life challenges, even those threatening her with domination and sublimation rituals [44]. Whereas the feminine hero has had to learn how to have a relationship to her body, the root of having what Jung called a Self ([18], p. 282). But as the feminine body can be interrupted through “punctuations” of menstruation, penetrative intercourse, becoming pregnant and breast feeding, rhythms resonating with vulnerability ([57], pp. 39–40) it can take time to make or find a Self if it has not been installed in early childhood through conducive social interactions [65] altering the lived experience of temporality. An unconscious relationship to her body difference from the masculine counterpart, including her vagina, womb, breasts and ovaries, may indicate her feelings are as an unknown aspect of self, therefore making her unavailable for relationship or procreative identity until how she appears to others, how she fears she will be used/not used, no longer betrays her loss of integrity through some kind of violation [66], even one of abjection, but emerges in synthesis toward the primary task of finding integrity within herself. The dichotomous struggle to achieve equality in political, social and economic fields between the sexes only to abandon the struggle in the sexual realm confuses the need to uphold sexual difference ([67], p. 139). In this dichotomous state lay the ingredients for an individuation process: psychic-physical tension with the potential for a union of opposites. “Creativity springs from the resolution and the reconciliation of opposing psychic forces within an individual” ([68], p. 83). This creativity is at the heart of the conclusion of the fairy tale Young-Eisendrath ([13], p. 18) draws from in considering the story of Sir Gawain and the Lady Ragnell ([13], p. 171 *n*.7), regarding what women really want: sovereignty over their own life. Here then lies the ethical methodological junction, where Feminist inspired Psychoanalytic and Feminist leaning Analytical Psychology join up to write an ethos for the use of intra-psychic and inter-subjectivity in research with female participants. The tension we are considering is when the body matters and when it does not.

“A complex… results from the blend of an archetypal core… and human experience particularly in the early years of life” ([69], p. 6). It is both these complex processes of psychic development this research seeks to bring together—*is delayed motherhood a revolt against domination of the biological imperative to reproduce in uncertain relationship to patriarchy?* This is an ethical question to do with non-normative sexual behavior, the place where Queer theory began its linguistic life before moving into gay and lesbian caucuses, Feminist politics upward to academic institutions, in parallel to rising awareness of AIDS [70] before turning on gender itself as an encasement of an “oppressive system of classification—both heterosexuality and homosexuality …as artificial categories” ([71], p. 29). 

Queer is evasive. “Just what ‘queer’ signifies or includes or refers to is by no means easy to say” ([72], p. 20). “Queer is a relation of resistance to whatever constitutes the normal” ([70], p. 99), the “open mesh of… excesses of meaning where the constituent elements of anyone’s gender, anyone’s sexuality aren’t made (or *can’t* be made) to signify monolithically” ([73], p. 8). Queer as a theoretical and *non-predictive-performative* condition may be emerging as a new signifier of normative behavior. In this way Queer undermines notions of feminine, masculine and eclipses both the conflict and union of opposites [49], something Jagose [70] describes as “holding open a space whose potential can never be known in the present” ([70], p. 107). Yet, “the conceptual slippage” in Butler’s theorizing of subject formation has resulted in “a lack of clarity… [regarding] the capacity for action held by subjects relative to the power that enables their existence in the first place” ([74], p. 28). The use of Queer Theory and consideration of Judith Butler’s later elucidation of a “‘third way’ between voluntarism and determinism” ([75], p. 291) is as much about *reconceiving* agency [76,77] as it is about holding an ethical position against pathologizing women who discover the need for motherhood and partnership later in life. Thus late motherhood is turning upside down Jung’s views of individuation in mid-life for women as a time of integrating the repressed masculine, a shift from an identity centered upon dependence and nurturer of others to one of agentic “embrace of one’s own development” ([13], p. 87). The task of procreative identity at mid-life appears as a new definition of a union of opposites, following the paradigm of first Adam then Eve. Unwittingly bio-technology has challenged, even re-arranged Jung’s life stages for women, though not the essence of his observation of the mid-life ‘calling’ to integrate what has been overlooked in the first half of life.

## 5. Concluding Thoughts

I did not enter into the research topic of a *midlife pregnant pause* [37] leading to late motherhood with Feminist intentions. Rather I had a Jungian perspective that cultural and collective complexes with hooks into personal complexes were getting in the way of the developmental aspect of achieving motherhood due to difficulties between the sexes. Delayed motherhood did not emerge as a Feminist issue until particular themes in regard to men in the form of absent or wayward fathers, overtly privileged brothers and betraying mothers began to surface. I came to see women as having to struggle with ‘indigenous’ cultural assumptions about their bodies being ordained for motherhood, extending a long period of adolescence while striving for accomplishment in the masculine world. Coming to motherhood was a reparative process the closer in age they came to embodying the stage of life known as ‘an older woman’ (Crone/Witch Archetype). In looking more closely at Psychoanalytically informed Feminist literature mainly written by women, I also discovered in Freud and Jung similar problems with the feminine, at different points in their professional development. These ‘problems’ mirrored the problems participants were implying with real male others regarding their own relationship with the feminine and integration of the masculine. In Feminist inspired analytic literature I found the body of the woman who had lost time during her most fertile years as context for the messages from the unconscious. In short, I came to see Jung and Freud as reproducing what has been long standing in civilization, a feminine split between denigration and idealization, and have used their words as evidence of patriarchal privilege, the screen through which each man analyzed female patients. It is my belief their work was the beginning of a longer work on the reproduction of misogynistic culture, with late motherhood appearing as a protection against androcentric interruption. Therefore, an ethical position to mutable and evolving expression and repression of the feminine necessitates in-depth understanding of these ingredients as alchemical products of intra-psychic and inter-subjective *primal material,* rather than constructing pathologies for non-participation in essentialist notions of feminine performance. Unconscious processes of the *embodied* feminine achieving late motherhood in mid-life emerged as a Feminist issue of power, control, defense, separation and repair. From this, a new union of epistemology and ethos has become impossible to ignore, in part because what is emerging in late motherhood is a different kind of mothering, on which rests the future of a different relationship to patriarchy.

## References

[1] Freud S., Strachey J., Gay P. (1933). New Introductory Lectures on Psycho-Analysis. The Complete Works of Sigmund Freud.

[2] Glocer Fiorini L., Glocer Fiorini L., Abelin-Sas Rose G. (2010). The Analyst’s Meta-Theories Concerning Sexual Difference and the Feminine. On Freud’s “Femininity”.

[3] Mitchell J. (1974). Psychoanalysis and Feminism.

[4] Appignanesi L. (2008). Mad, Bad and Sad: A History of Women and the Mind Doctors from 1800 to the Present.

[5] Chesler P. (2005). Women and Madness.

[6] Douglas C. (2000). Woman in the Mirror.

[7] Rowland S. (2002). Jung: A Feminist Revision.

[8] Pines D. (1993). A Woman’s Unconscious Use of Her Body.

[9] Swartz S. (2013). Feminism and psychiatric diagnosis: Reflections of a feminist practitioner. Fem. Psychol..

[10] Showalter E. (1992). Sexual Anarchy: Gender and Culture at the Fin de Siècle.

[11] Oppenheim J. (1991). “Shattered Nerve” Doctors, Patients, and Depression in Victorian England.

[12] Jung C.G., Douglas C. (1932). Visions. Notes of the Seminars Given in 1930–1934.

[13] Young-Eisendrath P. (1984). Hags and Heroes: A Feminist Approach to Jungian Psychotherapy with Couples.

[14] Hauke C., Alister I., Hauke C. (1998). Jung, Modernity and Postmodern Psychology. Contemporary Jungian Analysis.

[15] Chodorow J. (1999). The Reproduction of Mothering.

[16] Raphael-Leff J. (1984). Myths and modes of motherhood. Br. J. Psychother..

[17] Cowan L. Dismantling the Animus. The Jung Page. http://www.cgjungpage.org.

[18] Plaut F., Alister I., Hauke C. (1998). What do I Mean by Identity?. Jungian Analysis.

[19] Jung C.G., Read H., Fordham M., Adler G. (1995). Two Kinds of Thinking. The Collected Works of C.G. Jung.

[20] Samuels A., Shorter B., Plaut F. (1986). A Critical Dictionary of Jungian Analysis.

[21] Kulkarni C. (1997). Lesbians and Lesbianism.

[22] El Saffar R.A. (1994). Rapture Encaged The Suppression of the Feminine in Western Culture.

[23] Young-Eisendrath P., Toronto E.L.K., Ainslie G., Donovan M., Kelly M., Keiffer C., McWilliams N. (2013). The Female Person and How We Talk about Her. Psychoanalytic Reflections on a Gender-free Case: Into the Void.

[24] Young-Eisendrath P. (1997). Gender and Desire: Uncursing Pandora.

[25] Demaris D. (1988). Jung & Feminism Liberating Archetypes.

[26] Rowland S. (2005). Jung as a Writer.

[27] Rowland S. (2006). Jung, the trickster writer, or what literary research can do for the clinician. J. Anal. Psychol..

[28] De Beauvoir S. (1949). The Second Sex.

[29] Segal L. (1999). Why Feminism?.

[30] Raphael-Leff J. (2007). Femininity and its unconscious ‘shadows’: Gender and generative identity in the age of biotechnology. Br. J. Psychother..

[31] Harris A. (1991). Gender as contradiction. Psychoanal. Dialogues.

[32] Dimen M. (1991). Deconstructing difference: gender, splitting, transitional space. Psychoanal. Dialogues.

[33] Benjamin J. (1996). In defence of gender ambiguity. Gend. Psychoanal..

[34] Sweetnam A. (1996). The changing contexts of gender between fixed and fluid experience. Psychoanal. Dialog..

[35] Person E. (1999). The Sexual Century.

[36] Goldner V., Toronto E.L.K., Ainslie G., Donovan M., Kelly M., Kieffer C.C., McWilliams N. (2013). Ironic Gender, Authentic Sex. Psychoanalytic Reflections on a Gender-free Case: Into the Void.

[37] Barone-Chapman M., Jones R. (2010). Pregnant pause: Procreative desire, reproductive technology and narrative shifts at midlife. Body, Mind and Healing after Jung a Space of Questions.

[38] Braverton W.J., Alister I., Hauke C. (1998). The Collective Unconscious and Primordial Influences in Gender Identity. Contemporary Jungian Analysis.

[39] Woodman M. (1994). The Stillness Shall Be the Dancing: Feminine and Masculine in Emerging Balance.

[40] Smith D. (1990). Conceptual Practices of Power: A Feminist Sociology of Knowledge.

[41] Ussher J. (1991). Women’s Madness: Misogyny or Mental Illness?.

[42] Henderson J. (1984). Cultural Attitudes.

[43] Singer T., Kimbles S.L. (2004). The Cultural Complex Contemporary Jungian Perspectives on Psyche and Society.

[44] Benjamin J. (1988). The Bonds of Love: Psychoanalysis, Feminism, and the Problem of Domination.

[45] Graham Fuller V., Robinson H. (2003). Understanding Narcissism in Clinical Practice.

[46] Hillman J., Hillman J. (1972). On Psychological Femininity. The Myth of Analysis Three Essays in Archetypal Psychology.

[47] Freud S., Strachey J. (1937). Analysis Terminable and Interminable. The Standard Edition of the Complete Psychological Works of Sigmund Freud.

[48] Samuels A. (1989). The Plural Psyche.

[49] Barone-Chapman M., Mathers D. (2014). Sulphur Rises Through The Blackened Body. Alchemy and Psychotherapy.

[50] Samuels A. (1985). Jung and the Post-Jungians.

[51] Dinnerstein D. (1976). The Rocking of the Cradle and the Ruling of the World.

[52] Klein M. (1945). The Oedipus complex in light of early anxieties. Int. J. Psycho-Anal..

[53] Irigaray L., Gill G.G. (1985). Female Hom(m)osexuality: Speculum of the Other Woman.

[54] Chodorow N. (1989). Feminism and Psychoanalytic Theory.

[55] Orbach S., Eichenbaum L. (1982). Outside in inside out: A Feminist Psychoanalytic Approach to Women’s Psychology.

[56] Jung C.G., Read H., Fordham M., Adler G. (1959). The Syzygy: Anima and Animus. The Collected Works of C.G. Jung.

[57] Bianchi E., Gunkel H., Nigianni C., Soderback F. (2012). The Interruptive Feminine: Aleatory Time Feminist Politics. Undutiful Daughters New Directions in Feminist Thought and Practice.

[58] Gray F. (2008). Jung, Irigaray, Individuation Philosophy, Analytical Psychology, and the Question of the Feminine.

[59] Jung C.G., Read H., Fordham M., Adler G., Mc Guire W. (1970). The Personification of the Opposites. The Collected Works of C.G. Jung.

[60] Alexander Stewart J., Apperson V., Beebe J. (2008). Feminine Hero. The Presence of the Feminine in Film.

[61] Demme J. (1991). The Silence of the Lambs.

[62] Kristeva J. (1982). Powers of Horror An. Essay on Abjection.

[63] Douglas M. (1966). Purity and Danger.

[64] Walker B. (1983). The Woman’s Encyclopedia of Myth and Secrets.

[65] Zinkin L. (2008). Your Self: Did you find it or did you make it?. J. Anal. Psychol..

[66] Beebe J. (1992). Integrity in Depth.

[67] Schaeffer J., Fiorini L.G., Abelin-Sas Rose G. (2010). The Riddle of the Repudiation of Femininity. On Freud’s “Femininity”.

[68] Seligman E., Samuels A. (1985). The Half-Alive Ones. The Father: Contemporary Jungian Perspectives.

[69] Samuels A., Samuels A. (1985). Introduction. The Father: Contemporary Jungian Perspectives.

[70] Jagose A. (1996). Queer Theory: An Introduction.

[71] Young A., Gay and young (1992). Out of the Closets, into the Streets. Out of the Closets: Voices of Gay Liberation.

[72] Abelove H. (1993). From Thoreau to queer politics. Yale J. Crit..

[73] Sedgwick E.K. (1993). Tendencies.

[74] Brickell C. (2005). Masculinities, performativity, and subversion: A sociological reappraisal. Men Masculinities.

[75] Cadwallader J. (2009). How Judith Butler Matters?. Aust. Fem. Stud..

[76] Morrison T., Macleod C. (2013). A performative-performance analytical approach: Infusing Butlerian theory into the narrative-discursive method. Qual. Inq..

[77] Dow Magnus K. (2006). The unaccountable subject: Judith Butler and the social conditions of inter-subjective agency. Hypatia.

